# Muscle and adipose tissue morphology, insulin sensitivity and beta-cell function in diabetic and nondiabetic obese patients: effects of bariatric surgery

**DOI:** 10.1038/s41598-017-08444-6

**Published:** 2017-08-21

**Authors:** Stefania Camastra, Alessandra Vitali, Marco Anselmino, Amalia Gastaldelli, Rosario Bellini, Rossana Berta, Ilenia Severi, Simona Baldi, Brenno Astiarraga, Giorgio Barbatelli, Saverio Cinti, Ele Ferrannini

**Affiliations:** 1 Department of Clinical & Experimental Medicine, University of Pisa, Pisa, Italy; 2 Department of Experimental and Clinical Medicine-Center of Obesity, University of Ancona, Ancona, Italy; 3 Bariatric Surgery Unit, Santa Chiara Hospital, Pisa, Italy; 4 CNR Institute of Clinical Physiology, Pisa, Italy

## Abstract

Obesity is characterized by insulin-resistance (IR), enhanced lipolysis, and ectopic, inflamed fat. We related the histology of subcutaneous (SAT), visceral fat (VAT), and skeletal muscle to the metabolic abnormalities, and tested their mutual changes after bariatric surgery in type 2 diabetic (T2D) and weight-matched non-diabetic (ND) patients. We measured IR (insulin clamp), lipolysis (^2^H_5_-glycerol infusion), ß-cell glucose-sensitivity (ß-GS, mathematical modeling), and VAT, SAT, and *rectus abdominis* histology (light and electron microscopy). Presurgery, SAT and VAT showed signs of fibrosis/necrosis, small mitochondria, free interstitial lipids, thickened capillary basement membrane. Compared to ND, T2D had impaired ß-GS, intracapillary neutrophils and higher intramyocellular fat, adipocyte area in VAT, crown-like structures (CLS) in VAT and SAT with rare structures (cyst-like) ~10-fold larger than CLS. Fat expansion was associated with enhanced lipolysis and IR. VAT histology and intramyocellular fat were related to impaired ß-GS. Postsurgery, IR and lipolysis improved in all, ß-GS improved in T2D. Muscle fat infiltration was reduced, adipocytes were smaller and richer in mitochondria, and CLS density in SAT was reduced. In conclusion, IR improves proportionally to weight loss but remains subnormal, whilst SAT and muscle changes disappear. In T2D postsurgery, some VAT pathology persists and beta-cell dysfunction improves but is not normalized.

## Introduction

Obesity is associated with chronic low-grade inflammation in adipose tissue, which accompanies, and may contribute to the development of, insulin resistance and type 2 diabetes (T2D)^1^. In obese mice and humans, as hypertrophic adipocytes become insulin resistant, their lipolytic activity is accelerated; as a consequence, nonesterified fatty acids (NEFA) flux is partially shunted away from adipose tissue toward ectopic depots (in liver, muscle, and other organs). These changes in the metabolic phenotype are accompanied by an increased expression and release of inflammatory cytokines^2^, which further stimulate lipolysis. In addition, expanded adipose depots are infiltrated by macrophages and T cells, expressing high levels of inflammatory cytokines^3^. Macrophage infiltration is positively related to the size of adipocytes and coincides with the appearance of insulin resistance as macrophages alter the levels of insulin signalling molecules and GLUT4 and inhibit insulin action^4^.

In obese humans, adipocyte death is an accelerated phenomenon^5^. In both lean and obese mice over 90% of macrophages infiltrating the adipose tissue are found around dead adipocytes, forming characteristic structures termed ‘crown-like structures’ (CLS). Density of CLS is positively related to adipocyte size, independent of obesity status^6^. Adipose tissue from humans shows similar features^6^. Macrophages penetrate adipose tissue to remove remnants of dead adipocytes; due to the small size of macrophages and the time required for the dead adipocyte removal process, chronic low-grade inflammation is induced, similar to that observed in foreign body reactions^7, 8^. In mice, for adipocytes of the same size CLS density is higher in visceral adipose tissue (VAT), suggesting that adipocytes at this location are more fragile and reach a critical size that triggers death, termed the ‘critical death size’, earlier than adipocytes in subcutaneous adipose tissue (SAT)^9^.

These observations made in animal models and humans have led to the concept that the insulin resistance of obesity is a disease of the adipose tissue, which propagates to muscle (intramuscular lipid accumulation)^10^ and liver (steatosis)^11^. Implicit in this construct is that weight loss should cause regression of both the histologic and physiological abnormalities of the obese state. Despite the insulin resistance and low-grade inflammation, however, many obese subjects do not develop T2D, the emergence of which is marked and anteceded by a variable degree of beta-cell failure in both obese and lean individuals^12^. Whether T2D is associated with adipose tissue changes distinct from those of simple obesity is not clear given the frequent co-existence of the two conditions.

The first aim of the present study was to provide an integrated picture of both the histological (fat and muscle) and physiological (insulin resistance, lipolysis, subclinical inflammation, beta-cell function) abnormalities of obesity – and their relationships – in groups of non-diabetic or T2D obese individuals. To this end, we carried out morphologic (morphometry, immunohistochemistry, and electron microscopy) analyses of VAT, SAT, and skeletal muscle, measured insulin resistance (by the euglycaemic insulin clamp technique), beta-cell function (from a mixed meal test), and a range of inflammatory markers. The second aim was to test whether and to what extent the observed abnormalities resolve following weight loss, thereby verifying the assumption that fat accumulation is their prime cause. For this purpose, in the same subjects we exploited the potency of bariatric surgery (Roux-en-Y gastric bypass (RYGB)) to induce major weight loss and a stable reduced body weight 1 year after surgery, when the histological and physiological studies were repeated.

## Methods

### Subjects

We studied 13 morbidly obese patients with T2D and 15 gender-, and BMI-matched non-diabetic morbidly obese patients (ND). Diabetes was newly diagnosed in 3 patients, while in the other 10 patients known duration was 5.0 ± 1.6 years (range 1–10). Mean HbA_1c_ was 7.2 ± 0.5% (55.0 ± 4.6 mmol/mol) in the whole group of the T2D patients, antidiabetic treatment was diet alone in 5 patients, oral hypoglycaemic agents in 8 (metformin in 4, metformin plus a sulphonylurea in 3, pioglitazone in 1 patient). Eleven T2D and 8 ND participants were restudied 1 year after RYGB and received a repeat biopsy of subcutaneous adipose tissue and muscle. As a reference group for the metabolic variables, we used metabolic data from 611 non-diabetic, lean (BMI ≤ 25 kg/m^2^) men and women from the RISC Study^13^; glycerol turnover measurements were obtained in 8 of them. For comparison purposes of tissue analysis, fat biopsies were also available for 9 lean nondiabetic subjects (4 women and 5 men, age 53 ± 6 years, BMI = 23.6 ± 0.7 kg/m^2^) undergoing non-bariatric abdominal surgery; from these, subcutaneous fat was available for 6 subjects and visceral fat for 5 subjects. The study was approved by the Ethics Committee of the University of Pisa. The nature and purpose of the study were carefully explained to all subjects before they provided their written consent to participate to the study. All methods were performed in accordance with the relevant guidelines and regulations. Glucose flux and beta-cell function data from a larger group of surgical patients have been previously reported^14, 15^.

### Study design

At the time of study prior to surgery, body weight had been stable for at least six months and did not change between screening and study time. Antidiabetic therapy was discontinued one week before the metabolic study in the 8 diabetic patients on antihyperglycaemic treatment. Each metabolic study consisted of a euglycaemic hyperinsulinaemic clamp combined with the infusion of tracer glycerol^14^. On a different day at baseline and follow up, subjects received a mixed meal test (MMT, 75 g of glucose in 150 ml of water, 40 g of parmesan cheese, and one 50-g egg; 509 kcal, 16% protein, 28% fat, 56% carbohydrate)^15^.

### Surgical procedure and biopsies

RYGB was performed as described^14^. During the surgery, a few grams of omental (VAT) and subcutaneous (SAT) adipose tissue and of rectus abdominis muscle were excised and immediately fixed in 4% phosphate buffered formaldehyde solution, and sent for morphological evaluation. Likewise, adipose tissue samples were also obtained in lean controls during non-bariatric abdominal surgery. In the patients that were restudied 1 year after RYGB, percutaneous biopsies of subcutaneous fat and muscle were performed. Briefly, participants were instructed not to perform strenuous physical exercise for 48 hours before the biopsy procedure. Under local anaesthesia (10 ml carbocaine), tissue specimens were obtained through a small incision of the left abdominal mid-quadrant (at the same level of the previous laparoscopic access).

### Analytical procedures

Plasma glucose was measured by the glucose oxidase technique (Analox GM-9), plasma insulin and C-peptide by electrochemiluminescence (on a COBAS e 411 instrument, Roche, Indianapolis, USA). Plasma triacylglycerols and serum high-density lipoprotein (HDL) cholesterol were assayed in duplicate by standard spectrophotometric methods on a Synchron Clinical System CX4 (Beckman Instruments, Fullerton, USA). Plasma ^2^H_5_-glycerol enrichment was measured by gas chromatography/mass spectrometry (GCMS), as described previously^16^.

### Data analysis

Whole-body fat-free mass was estimated by electric bioimpedance on a Tanita scale; of note, this method has been validated in very obese individuals against the deuterated water technique^17^. Lipolysis was estimated as the glycerol rate of appearance (RaGly, in µmol.min^−1^), and calculated using steady-state equations for the fasting state and Steele’s equations during the clamp, as previously described^14^. An index of adipose tissue insulin resistance (AT-IR) was calculated as the product of RaGly (normalized per kg of fat mass) and fasting plasma insulin concentration^18^.

Insulin-stimulated glucose disposal was expressed per kg of fat-free mass (FFM) (M, µmol.min^−1^.kg_FFM_^−1^); insulin sensitivity was indexed as the ratio of M to the steady-state plasma insulin concentrations (M/I, in µmol.min^−1^.kg_FFM_^−1^.pM^−1^)^14^.

Beta-cell function was quantitated by mathematical modeling of the plasma C-peptide response to the MTT, as described^19, 20^. The test, which allows to obtain an estimate of the principal parameters of beta-cell function after a physiological stimulus, is better tolerated than oral glucose postsurgery. The main parameter of the model is beta-cell glucose sensitivity ß-GS), which is calculated as the mean slope of the dose-response function (*i*.*e*., the relationship between insulin secretion rates and plasma glucose concentrations during corresponding times of the MMT). The model also yields a measure of the total amount of insulin released over the 5 hours of the meal^15^.

### Tissue analysis

Samples of skeletal muscle were dehydrated in ethanol, postfixed in osmium, and paraffin-embedded^21^. Transverse sections were stained with hematoxylin-eosin and analyzed for the presence of lipid droplets in the perivascular space (perivascular lipid), between fibers (interfibrillar lipid), and inside fibers (intramyocellular lipid). In each location, lipid content was scored as follows: 0, none; 0.5, just distinguishable, with focal distribution; 1, detectable in restricted areas of the parenchyma but in scarce amount; 2, well evident in different areas of the parenchyma; and 3, numerous and/or voluminous, generally confluent droplets in diffuse areas of the parenchyma. The quantitative assessment was carried out by three authors (A.V., I.S. and G.B.) who were blinded to the participant group and metabolic data; the results were tested in repeated measurements of the same specimen and were found to be reproducible within 10%. This method was also used in a previous paper^21^.

### Light microscopy and immunohistochemistry of adipose tissue

For light microscopy and immunohistochemistry, tissue fragments were paraffin embedded and sectioned for morphometric evaluations; hematoxylin/eosin staining was used to assess morphology. Other selected sections were stained by immunohistochemistry (with the use of the Mac-2 and CD68 antibody to reveal the presence of macrophages and CLS) using a Vectastain ABC Kit peroxidase labeled (Vector Laboratories) and enzyme localization by DAB substrate^22^.

### Electron microscopy

Subcutaneous and omental fat samples obtained during surgery and subcutaneous fat from biopsies were also analyzed by transmission electron microscopy. Small fragments of tissue were fixed in 2% glutaraldehyde and 2% paraformaldehyde in 0.1 M phosphate buffer, pH 7.4, for 4 hours, postfixed in 1% osmium tetroxide, and embedded in an Epon-Araldite mixture. Semi-thin sections (2 µ) were stained with toluidine blue, and thin sections were obtained with an MT-X ultratome (RMC, Tucson, AZ), stained with lead citrate. At least three thin section for each patient were examined in a blinded way with a CM10 transmission electron microscope (Philips, Eindhoven, The Netherlands)^22^. Morphometric evaluations were performed using an image analysis software (Lucia IMAGE version 4.82, Laboratory Imaging). Five patients in each condition were examined. Ten electron micrographs of each adipose depot of each patient were taken to calculate cytoplasmic and basal membrane thickness (expressed in µm), size (µm^2^), and density (number of mitochondria in 10 µm^2^) of mitochondria.

### Morphometry

Tissue sections was observed with a Nikon Eclipse E800 light microscope using a 20 objective, and digital images were captured with a DXM 1200 camera. Crown-like structure (CLS) density (CLS per 104 adipocytes), adipocyte surface area, and adipocyte volume was determined by the Nikon Lucia IMAGE (v. 4.61) image analysis software^22^.

### Statistical analysis

Data are given as mean ± SEM or, if non-normally distributed, as median and [interquartile range]. The Mann-Whitney U test was used to compare group values, paired group values were analyzed by Wilcoxon sign rank test. Treatment-induced changes were tested by repeated-measure ANOVA, with group (ND or T2D) as a fixed effect; for this analysis, non-normally distributed variables were log-transformed. Simple associations were tested by calculating the Spearman rank correlation coefficient (ρ). Statistical analyses were carried out using Stat View 5.0 software; a *p* value ≤ 0.05 was considered significant.

## Results

### Baseline

T2D patients were older than ND subjects but had similar body weight and composition. Both T2D and ND subjects were markedly insulin resistant (M/I) as compared with historical lean controls^13^ (Table 1). T2D patients had fasting hyperglycaemia, lower total insulin secretion rate, and profoundly impaired beta-cell glucose sensitivity (ß-GS) compared with ND (Table 1). Neither adiponectin nor cytokines (MCP-1, IL-6, and TNFα) differed between the two groups. All metabolic parameters of the surgical patients were significantly altered in comparison to the group of historical controls, with the exception of ß-GS which was preserved in non-diabetic obese patients.

**Table 1:** Baseline anthropometric and metabolic parameters in nondiabetic (ND) and diabetic (T2D) surgical patients and in non-obese control subjects (Ctrls)^§^.

	ND	T2D	p*	Ctrls
Number (F/M)	15 (14/1)	13 (9/4)	ns	611 (405/206)
Age (years)	41 ± 3	50 ± 2	0.01	43 ± 1
BMI (kg.m^−2^)	51.0 ± 1.6	50.0 ± 2.2	ns	22.4 ± 1.8°
FM (%)	52 ± 1	47 ± 2	ns	24 ± 8°
FM (kg)	72 ± 4	63 ± 5	ns	16 ± 5°
FFM (kg)	67 ± 3	71 ± 5	ns	49 ± 9°
Waist circumference (cm)	133 ± 3	141 ± 7	ns	78 ± 9°
Fasting glucose (mmol/L)	5.5 ± 0.1	9.1 ± 0.8	<0.0001	4.9 ± 0.5°
Fasting insulin (pmol/L)	138 [72]	137 [115]	ns	23 [16]°
Plasma NEFA (mmol/L)	0.58 [0.25]	0.67 [0.14]	ns	0.51 [0.26]°
Total cholesterol (mmol/L)	4.5 ± 0.2	4.6 ± 0.3	ns	4.8 ± 0.9
Triacylglycerols (mmol/L)	1.2 [0.5]	1.3 [0.7]	ns	0.8 [0.5]°
HDL-cholesterol (mmol/L)	1.2 ± 0.1	1.1 ± 0.1	ns	1.6 ± 0.4°
Adiponectin (ng/ml)	6.1 ± 0.7	6.7 ± 1.0	ns	9.5 ± 4.1°
MCP-1 (pg/ml)	235 [154]	265 [120]	ns	nd
IL-6 (pg/ml)	3.66 [2.19]	5.77 [3.54]	ns	0.86 [1.6]°
TNFa (pg/ml)	1.45 [0.61]	1.17 [0.46]	ns	0.75 [0.31]°
M/I (nmol^.^min^−1.^kg_FFM_^−1.^pM^-1^)	31 [22]	25 [31]	ns	154 [83]°
RaGly (µmol^.^min^−1^)	354 [92]	349 [91]	ns	113 [86]°
AT-IR (mmol^.^min^−1.^kg_FM_^−1.^pM)	0.54 [0.63]	0.83 [0.68]	ns	0.30 [0.16]°
Mean Glucose (mmol/L)	6.0 ± 0.2	9.8 ± 1.0	<0.0001	6.3 ± 0.1°
ß-GS (pmol^.^min^−1.^m^−2.^mM^−1^)	128 [42]	21 [24]	<0.0001	125 [86]°
Total insulin output (nmol^.^m^−2^)	97 [41]	50 [45]	0.004	37 [15]°
Fasting ISR (pmol^.^min^−1.^m^−2^)	144[103]	99 [100]	ns	59 [26]°

^§^Entries are mean ± SEM or median [interquartile range]; BMI = body mass index; FM = fat mass; NEFA = nonesterified fatty acids; MCP-1 = monocyte chemoattractant protein; IL-6 = interleukin-6; TNFα = tumor necrosis factor-α; M/I = insulin-mediated glucose disposal rate; RaGly = glycerol rate of appearance; AT-IR = adipose tissue insulin resistance index; Mean Glucose = calculated from the glucose AUC during MTT; ß-GS = beta-cell glucose sensitivity; ISR = insulin secretion rate; nd = not determined.

*T2D *vs* ND by Mann Whitney test.

°p ≤ 0.05 for the difference between controls (Ctrls) and the combined ND and T2D group.

### Light microscopy

**Figure 1:**
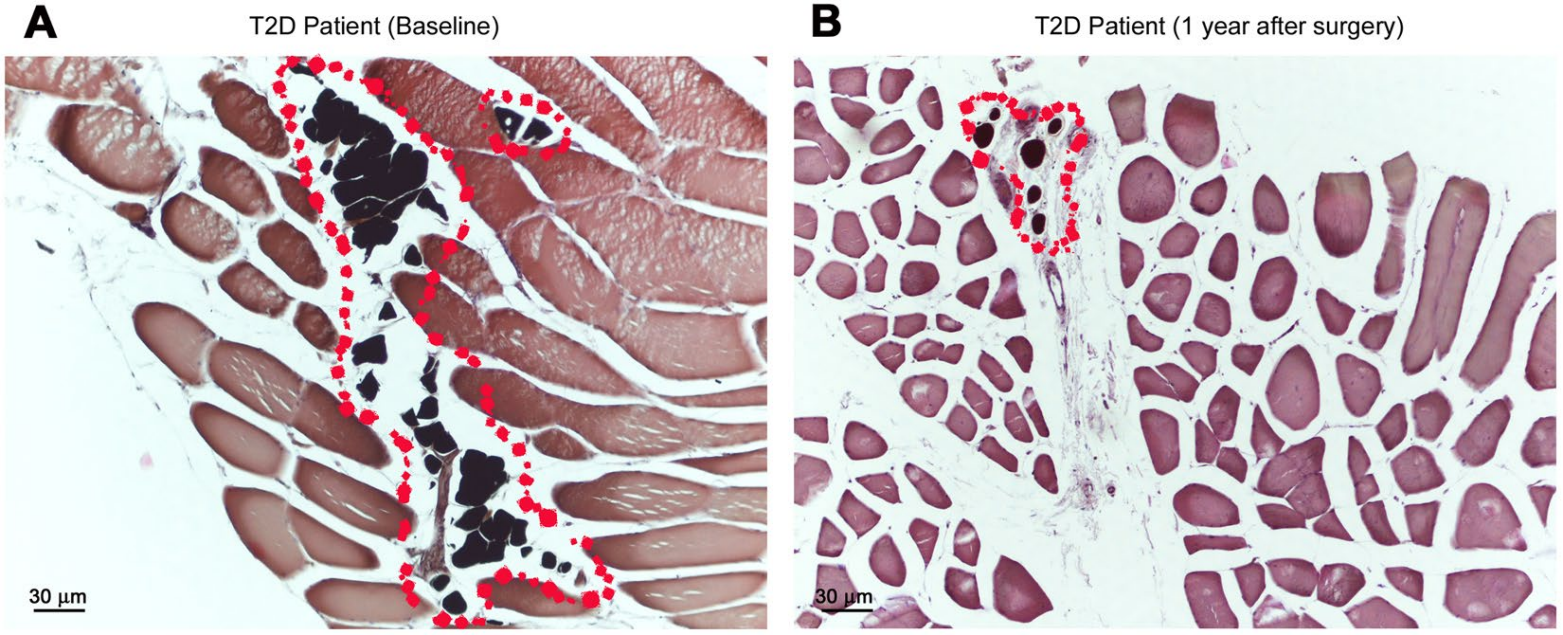
Skeletal muscle from a diabetic (T2D) patient pre (**A**) and post (**B**) bariatric surgery. A reduction of intermyocellular fat is evident (red dots).

**Figure 2:**
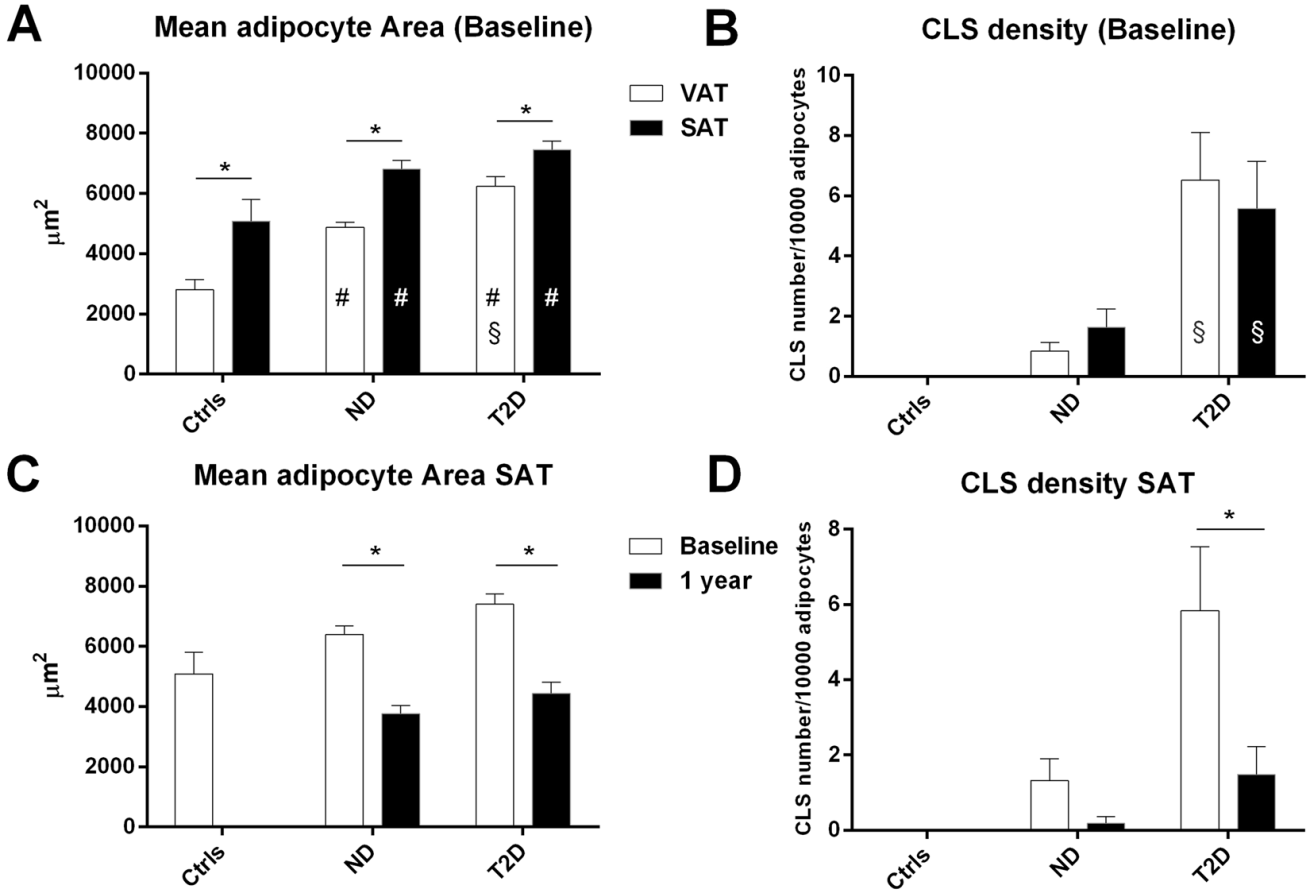
Mean adipocyte area (**A**) and crown-like structure density (CLS number/104 adipocytes, (**B**)) from lean controls (Ctrls), ND and T2D patient biopses of visceral (VAT) and subcutaneous (SAT) depots before surgery (Baseline). In **C** and **D**, the same parameters have been shown in subcutaneous adipose tissues from ND and T2D patient biopsies before (Baseline) and post-surgery (1 year after surgery). In (**A** and **B**) ^(#)^p < 0.05 by Mann Whitney U test for comparison of ND and T2D *vs* Ctrls; ^(§)^p < 0.05 by Mann Whitney U test for comparison of T2D *vs* ND; and ^(*)^p < 0.05 by Wilcoxon sign rank test to compare VAT *vs* SAT in each group. In C and D ^(*)^p < 0.05 for the changes at 1 year *vs* baseline by Wilcoxon sign rank test.

Fat accumulation in *rectus abdominis* muscle was similar between T2D and ND patients in the perivascular and interfibrillar areas (Table 2). In contrast, intramyocellular fat was more abundant in T2D than ND patients (Fig. 1A). In SAT, the adipocyte area was similar in T2D and ND patients (Fig. 2A), and larger than in the lean controls (adipocyte area SAT in lean controls group 5349 [2948] µm^2^, p < 0.04 *vs* ND and p < 0.01 T2D). In VAT, the adipocyte area was smaller than in SAT, was larger in T2D than ND patients (Fig. 2A) and expanded as compared to lean controls (adipocyte area VAT of lean controls group 2575 [807]µm^2^, p < 0.04 *vs* ND and p < 0.01 T2D). In both depots, CLS density was higher in T2D than ND patients (Fig. 2B).

**Table 2:** Changes in muscle histology (semi-quantitative analyses)^§^.

	Baseline		Effect of surgery			
	ND	T2D	ND pre	ND post	T2D pre	T2D post
Number (F/M)	15 (14/1)	13 (9/4)	8 (7/1)	8 (7/1)	11 (7/4)	11 (7/4)
Perivascular muscle fat (units)	1.25 [1.75]	2.00 [2.50]	2.25 [1.50]	1.50 [1.88]	2.00 [2.25]	1.00 [0.75]
Interfibrillar muscle fat (units)	1.50 [1.44]	2.00 [1.00]	1.50 [1.00]	0.75 [0.94]	2.00 [1.00]	0.75 [0.88]*
Intramyocellular fat (units)	0.00 [0.44]	1.00 [0.50] °	0.12 [0.50]	0.00 [0.00]	1.00 [0.50]	0.00 [0.25]*

^§^entries are median [interquartile range].

°p ≤ 0.01 T2D *vs* ND by Mann Whitney test.

*p ≤ 0.05 for the changes at 1 year *vs* baseline by Wilcoxon sign rank test.

**Figure 3:**
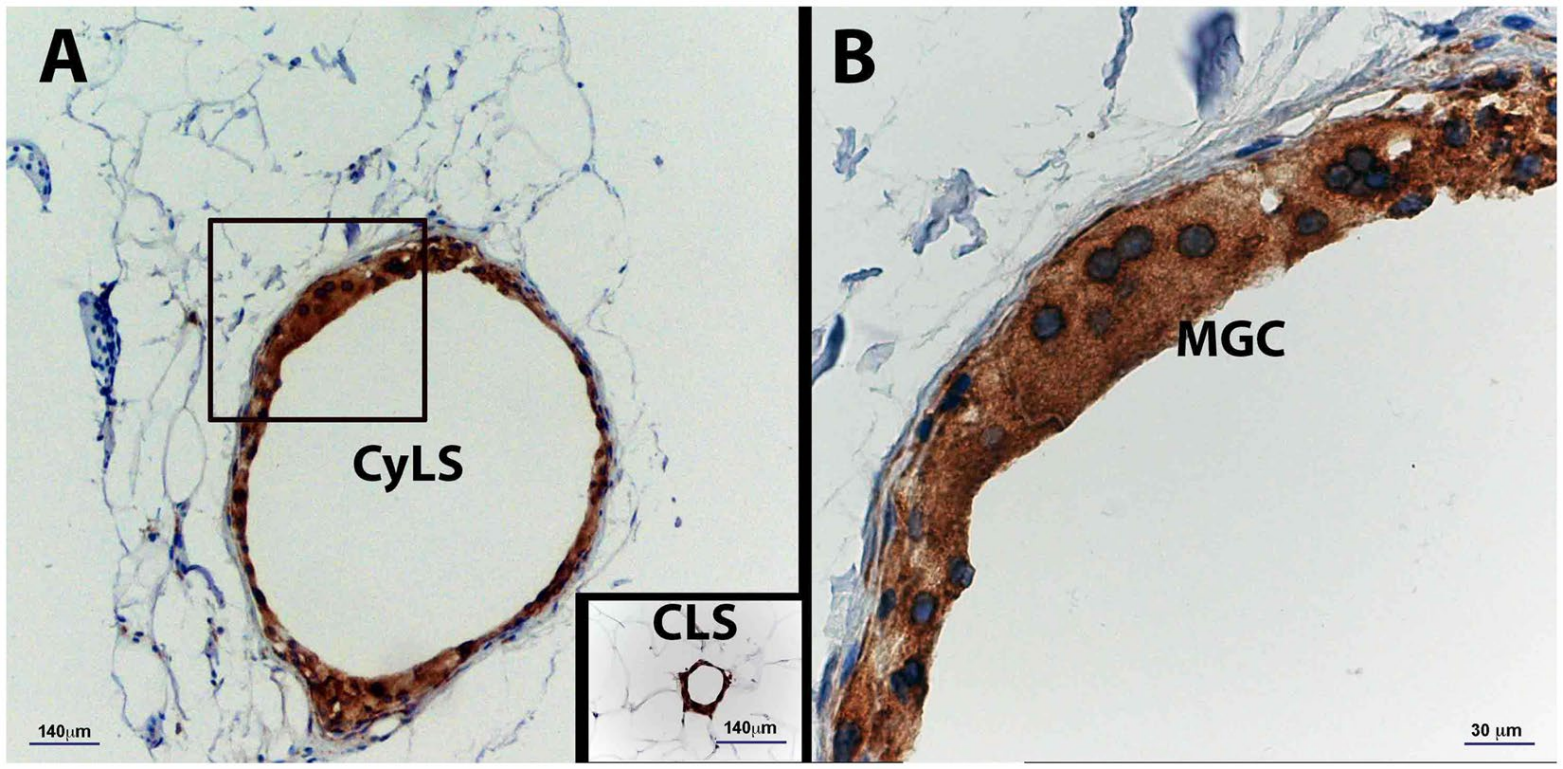
Light microscopy of subcutaneous adipose tissue (SAT) of an obese patient with type 2 diabetes. Immunohistochemistry for CD68. (**A**) Cyst-Like-Structure (Cys-LS) formed by CD68 immunoreactive macrophages and multinucleated giant cells (MGC) surrounding fat, amidst hypertrophic adipocytes. Note the giant size of Cys-LS in comparison with hypertrophic adipocytes and the largest CLS found in the same patient. (**B**) Magnification of the inset in **A**.

In SAT of two T2D patients we found structures never described previously. They had the general structural characteristics of CLS (*i*.*e*., roundish, perilipin-negative central lipid droplet) (Fig. 3A) with CD68-immunoreactive macrophages and giant CD68-immunoreactive multinucleated cells surrounding the lipid core (Fig. 3B). Their size was ~10-fold that of the largest CLS and largest adipocyte ever described (Fig. 3A inset). We named these structures Cyst-Like-Structures (CyLS). In these 2 individuals, the size of VAT and SAT adipocytes exceeded by ~30% that of the average adipocyte area of the other 26 subjects in the current series.

### Electron microscopy

#### VAT in ND patients

**Figure 4:**
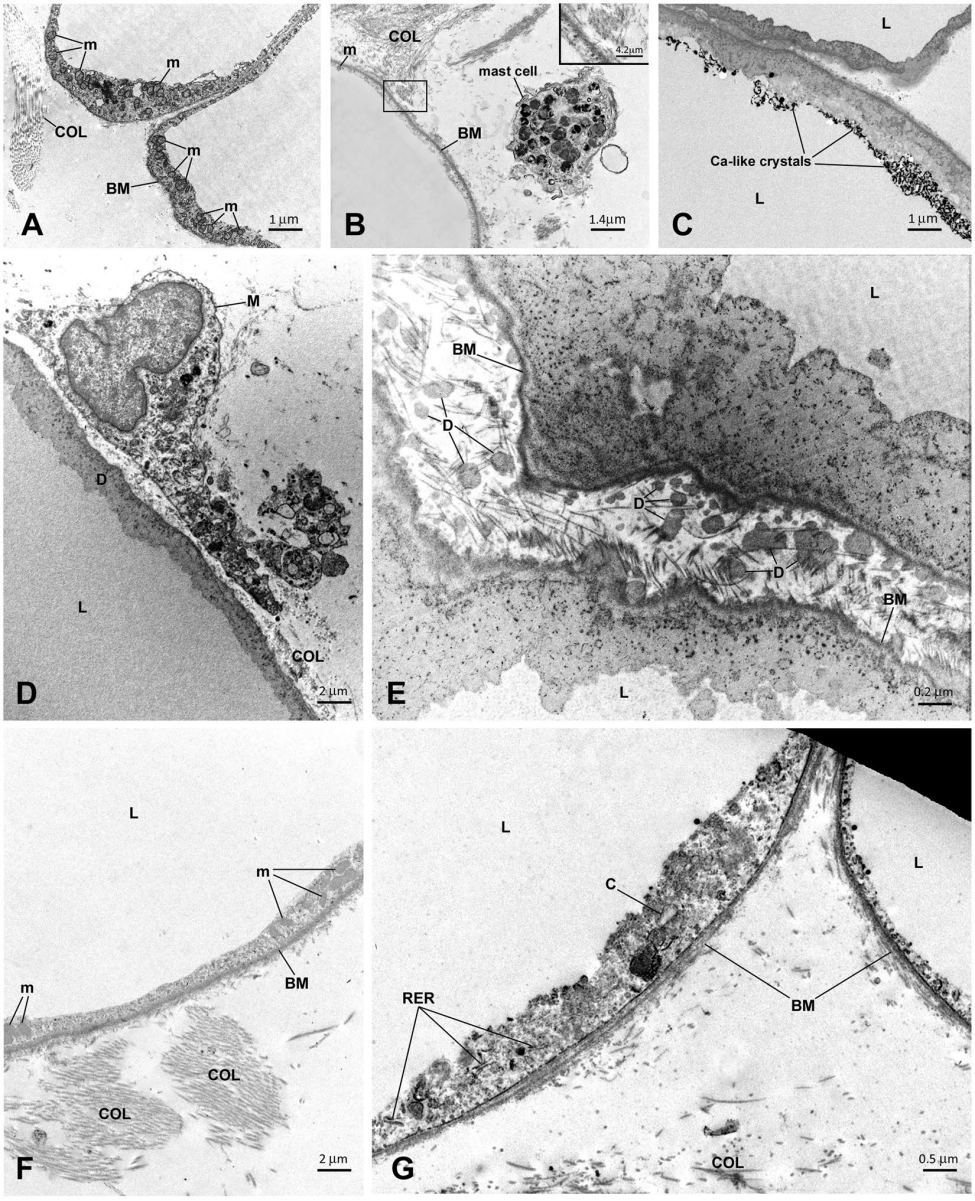
In **A**,**B** and **C** electron microscopy of visceral fat. (**A**) Visceral fat of a lean control subject. (**B**) Visceral fat of an obese non-diabetic (ND) patient. Note the different thickness of the cytoplasmic rim, small and rare mitochondria and abundant collagen (enlarged in the inset) in the obese patient. A mast cell is close to the adipocyte of the obese patient. (**C**) Visceral fat of a T2D patient. The cytoplasm of two degenerating adipocytes is visible. Note the presence of calcium-like crystals in one adipocyte. In **D** and **C** electron microscopy of subcutaneous adipose tissue (SAT) of two obese patients with type 2 diabetes. (**D**) Degenerative signs (D) and a close-by macrophage in the interstitium (M). (**E**) The cytoplasm of two degenerating adipocytes is visible. The lipid droplet (L) and basal membrane (BM) are still distinguishable, but the cytoplasm and organelles are not recognizable and are mixed with a dark material that seems to overlay the basal membrane and occupy the interstitium in the form of irregular dots (D) with the same electrondensity of the degenerating cytoplasm. Overall, the fine morphology is compatible with that of degenerating adipocytes (Cinti S *et al*.^6^. In **F** and **G** electron microscopy of subcutaneous adipose tissue (SAT) of two obese patients with type 2 diabetes post-surgery. (**F**) The cytoplasmic rim is thicker than pre-surgery (*cfr*, **E**) and clusters of mitochondria (m) are visible. BM = basal membrane, COL = collagen fibrils. (**G**) In this adipocyte (left), signs of stress were present: dilated rough endoplasmic reticulum (RER), cholesterol crystals (C), and thick basal membrane (BM). L = lipid droplets, m = mitochondria, BM = basal membrane, COL = collagen fibrils. 196 × 297 mm (150 × 150 DPI).

In VAT of ND subjects, adipocytes displayed a thin rim (ND = 0.48 [0.16] µm *vs* Ct = 0.80 [0.27] µm, *p* = 0.01, Fig. 4A), few small mitochondria and frequent lipofuscins; abundant collagen fibrils were observed in close association with the basal membrane and in the interstitium (Fig. 4B). Rare cytoplasmic crystals, and dilated rough endoplasmic reticulum (RER) were also found. Degenerating adipocytes (in line with the presence of perilipin-negative adipocyte-like cells by immunohistochemistry) and extrusion of lipid droplets in the interstitium were frequently found. Basal membranes were often thickened and highly irregular. Mast cells were found in close proximity of adipocytes and sometimes surrounding intact adipocytes to form clustering arrays.

#### VAT in T2D patients

In VAT of T2D patients, additional changes were calcium-like crystals in the cytoplasm of some cells (Fig. 4C), macrophages in close proximity to adipocytes (also detected by CD68 immunohistochemistry), and neutrophils in the interstitium and blood capillaries, likely expression of parenchymal inflammation.

#### SAT in ND patients

In SAT of ND patients, adipocytes had very thin cytoplasm (0.37 [0.45] µm, *i*.*e*., about 25% thinner than that of VAT adipocytes in line with their larger size), with very small (ND = 0.06 [0.01] µm^2^ *vs* Ct = 0.14 [0.06] µm^2^, *p* = 0.01), rare (ND = 1.08 [0.57]/10 µm^2^ *vs* Ct = 4.22 [2.09]/10 µm^−2^, *p* = 0.01) mitochondria. Organelles were very rarely visible, and often degenerative signs were observed. Basal membranes were often thickened (ND = 0.118 [0.018] µm *vs* Ct = 0.073[0.009] µm, *p* = 0.01) and sometimes duplicated. In the interstitium, free lipid droplets, and macrophages could be found, and fibrosis was less pronouced than in VAT.

#### SAT in T2D patients

In SAT of T2D patients, rare crystals and glycogen accumulation were observed in the cytoplasm of some adipocytes. There were frequent degenerating adipocytes (Fig. 4D,E), and intravascular parenchymal inflammation, with neutrophils, macrophages, and free lipids in the interstitium. Inflammatory cells and fibrosis as well as irregular basal membranes (T2D = 0.111 [0.025] µm *vs* Ct = 0.073 [0.009] µm, *p* = 0.01) were more pronounced than in lean patients.

### After surgery

One year after surgery, patients in both groups had lost an average 33% of their initial body weight and most metabolic parameters had improved significantly (Supplemental Table S1). Three patients experienced dumping like symptoms (nausea, sweating, tachycardia) after meal ingestion. In the T2D group, all patients were off antidiabetic treatment, and mean HbA_1c_ was 5.5 ± 0.2% (36.6 ± 1.7 mmol/mol) (*p* = 0.01 *vs* baseline). Adiponectin levels were increased and IL-6 decreased, whereas TNFα was unchanged. Insulin sensitivity (M/I) was significantly improved compared to baseline in both groups; in contrast, beta-cell glucose sensitivity was slightly reduced in ND and much improved – though not normalized – in T2D.

### Light microscopy

**Figure 5:**
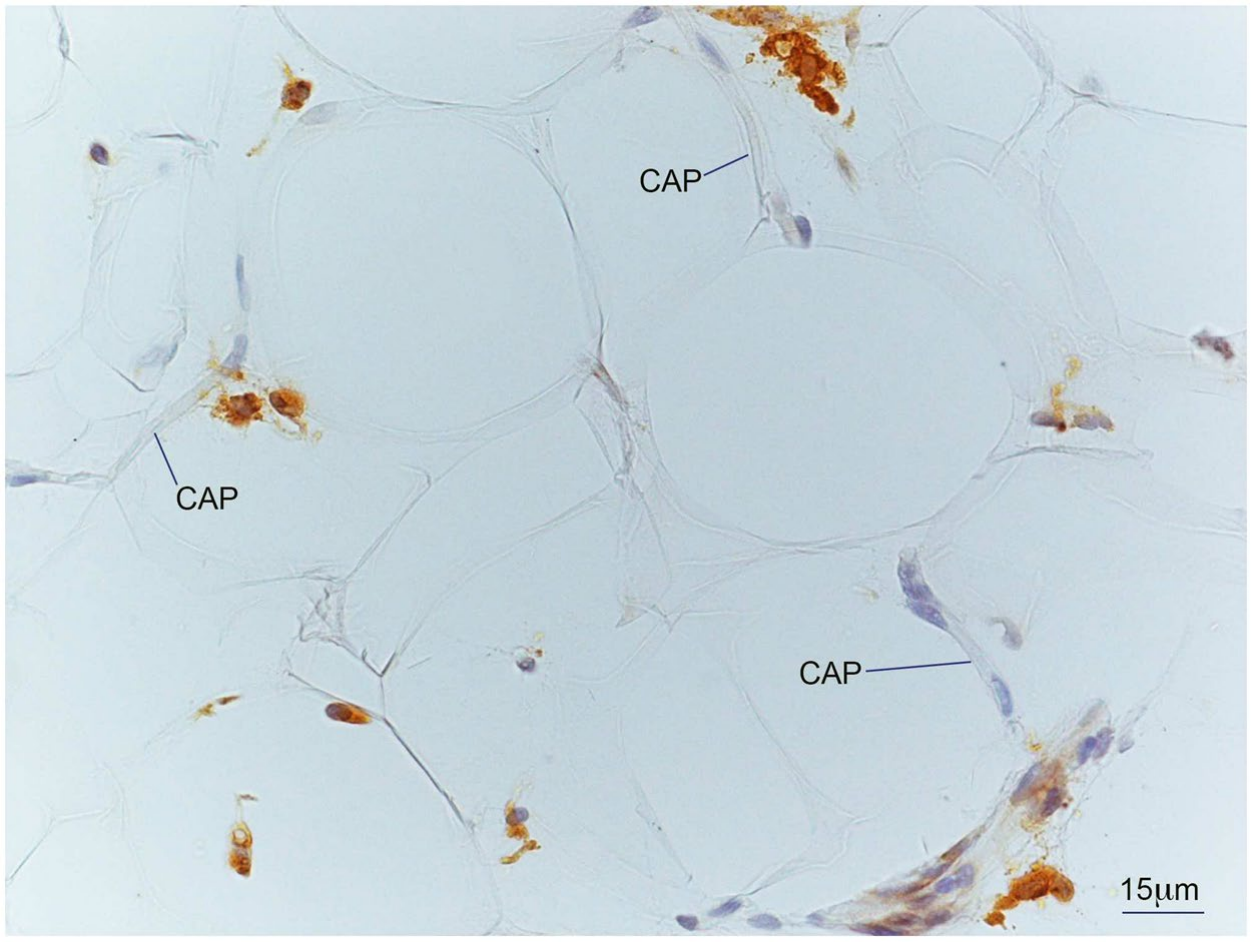
Light microscopy of subcutaneous adipose tissue (SAT) of an obese patient with type 2 diabetes post-surgery. Immunohistochemistry for CD68. Several CD68 immunoreactive macrophages (brown) are present among unstained adipocytes and capillaries (CAP).

In *rectus abdominis*, fat deposition tended to be reduced in all locations, perivascular, interfibrillar and intramyocellular in both ND and T2D (Table 2). In particular, intramyocellular fat had virtually disappeared in both ND and T2D patients (Fig. 1B). In SAT, adipocyte area was markedly reduced in both groups, and was no longer expanded as compared to lean controls (Fig. 2C). CLS density in SAT was reduced in both groups (Fig. 2D). Sparse (not organized in CLS) CD68-immunoreactive macrophages were still present in the tissue of most patients, especially of T2D patients (Fig. 5).

### Electron microscopy

In SAT of both ND (n = 6) and T2D (n = 8) individuals, adipocytes showed a thicker cytoplasmic rim (before *vs* after surgery: ND = 0.37 [0.45] *vs* 0.58 [0.26] µm, *p* = ns; T2D = 0.44 [0.27] *vs* 0.58 [0.10] µm, *p* = 0.04) containing more mitochondria (before *vs* after surgery: ND = 1.08 [0.57]/10 µm^2^ *vs* 1.98 [0.92]/10 µm^2^, *p* = 0.04; T2D = 1.54 [1.11]/10 µm^2^ *vs* 3.11 [1.99]/10 µm^2^, *p* = ns) (Fig. 4F). Dilated RER, glycogen accumulation, cholesterol-like cristals and thickened basal membrane (before *vs* after surgery: ND = 0.118 [0.018] µm *vs* 0.123 [0.011] µm, *p* = ns; T2D = 0.111 [0.025] *vs* 0.103 [0.026] µm, *p* = ns) were still present only in some cells of both ND and T2D patients (Fig. 4G).

#### Correlations

Most of the baseline measures were intercorrelated both before and after surgery; the pattern of statistically significant associations is summarized in Table 3. In brief, a higher BMI or waist circumference was positively associated with larger adipocytes and more numerous CLS in both SAT and VAT, more muscle fat, and raised circulating IL-6 concentrations (waist *p* = 0.0008, *r* ^2^ = 0.35; BMI *p* = 0.0001, *r* ^2^ = 0.30). In turn, expansion of SAT depots was associated with lower adiponectin levels (*r* ^2^ = 0.32), and with muscle (M/I, *r* ^2^ = 0.51) and adipose tissue insulin sensitivity (AT-IR, *r* ^2^ = 0.34). Muscle and adipose tissue insulin sensitivity was related to perivascular fat (M/I *r* ^2^ = 0.12; AT-IR *r* ^2^ = 0.26) and interfibrillar muscle fat (M/I *r* ^2^ = 0.13). Increased VAT adipocyte area was related to more CLS both in VAT (*p* = 0.005, *r* ^2^ = 0.30) and in SAT (*p* = 0.008, *r* ^2^ = 0.35) and more intramyocellular fat (*r* ^2^ = 0.36). Furthermore in baseline condition, ß-GS was inversely related to VAT adipocyte area (*r* ^2^ = 0.34) and VAT CLS density (*p* = 0.02, *r* ^2^ = 0.25) and to intramyocellular fat (*p* = 0.006, *r* ^2^ = 0.37). These three variables explained 64% of the variation of ß-GS (*p* = 0.004, *r* ^2^ = 0.64) before surgery. All the described relationships were mantained after adjusting for age and sex in a multivariate analysis.

**Table 3:** Correlation among clinical, histological, and metabolic parameters in the combined ND and T2D groups.

	BASELINE		BEFORE AND AFTER SURGERY													
	Adipocyte area VAT		CLS VAT		Adipocyte area SAT		CLS SAT		Perivascular muscle fat		Interfibrillar muscle fat		Intramyocellular fat		Adipocyte mitochondria size	
	r	*p*	r	*p*	r	*p*	r	*p*	r	*p*	r	*p*	r	*p*	r	*p*
Adipocyte area SAT	0.76	<0.0001		ns	—	—	0.60	<0.0001		ns	0.54	0.0001	0.57	0.0006	−0.36	0.01
CLS SAT	0.59	0.008	0.61	0.006	0.60	<0.0001	—	—		ns		ns	0.39	0.03		ns
Perivasc. muscle fat		ns	0.47	0.04		ns		ns	—	—	0.43	0.007		ns		ns
Interfibrill. muscle fat		ns		ns	0.54	0.001		ns	0.43	0.007	—	—	0.50	0.003		ns
Intramyocellular fat	0.60	0.005		ns	0.57	0.0006	0.39	0.03		ns	0.50	0.003	—	—		ns
BMI		ns		ns	0.89	<0.0001	0.50	0.001		ns	0.45	0.004	0.42	0.008	−0.61	<0.0001
Waist circumference	0.59	0.01	0.54	0.03	0.86	<0.0001	0.61	0.001	0.44	0.03	0.44	0.03	ns	ns	−0.41	0.004
Fasting glucose		ns	0.60	0.002	0.44	0.003	0.36	0.04	0.36	0.04		ns	0.35	0.04		ns
Fasting insulin		ns		ns	0.70	<0.0001	0.52	0.0008	0.46	0.004		ns	0.36	0.03	−0.20	0.049
HbA1c		ns	0.65	0.02	0.54	0.02	0.57	0.02		ns		ns		ns		ns
Mean Glucose	0.43	0.04	0.53	0.007	0.40	0.009	0.57	0.0003		ns	0.41	0.01		ns		ns
Adiponectin		ns		ns	−0.56	0.0002	−0.39	0.02		ns		ns		ns	0.54	0.0004
MCP1		ns		ns	0.35	0.03	0.37	0.03		ns		ns	0.40	0.02		ns
IL-6		ns		ns	0.50	0.001		ns	0.41	0.02		ns	0.40	0.02		ns
M		ns	−0.42	0.03	−0.62	<0.0001	−0.42	0.009	−0.35	0.03		ns		ns		ns
M/I		ns		ns	−0.72	<0.0001	−0.43	0.008	−0.35	0.03	−0.36	0.03		ns	0.38	0.004
AT-IR		ns		ns	0.58	<0.0001	0.35	0.04	0.51	0.001		ns		ns		ns
ß-GS	−0.58	0.004	−0.50	0.02		ns	−0.43	0.01		ns		ns	−0.48	0.004		ns
Fasting ISR		ns		ns	0.54	0.0002	0.45	0.006	0.41	0.002		ns		ns		ns

## Discussion

The main findings of this study are: (a) the alterations of fat and skeletal muscle morphology of the obese correlate well with the insulin resistance and associated metabolic abnormalities that characterize this condition; (b) both VAT and SAT adipocytes exhibit more marked histologic abnormalities in T2D than ND subjects; (c) VAT histology is associated with beta-cell dysfunction in T2D; and (d) following major weight loss, tissue histology and metabolic function improve consensually; however, in T2D incomplete recovery of beta-cell function suggests the presence of an inherent secretory defect. These findings require specification.

Firstly, the baseline data obtained before surgery recapitulate the key metabolic findings of obesity and T2D. Thus, our patients showed insulin resistance of glucose metabolism and lipolysis, hyperinsulinemia and insulin hypersecretion, dyslipidemia, reduced levels of adiponectin, and raised circulating levels of markers of inflammation. In the T2D patients, beta-cell glucose sensitivity was severely impaired in comparison with the weight-matched ND subjects. Secondly, adipocyte size was clearly expanded in both subcutaneous and visceral depots, with evidence of cell stress, degeneration and necrosis, in line with recent observations in murine obese adipocytes^23^. The significant association of adipocyte size with increased lipolysis and depressed insulin action (*ρ* = 0.73 for both, *p* < 0.0001) confirms the pathogenic potential of adipocyte hypertrophy in humans^24–26^. This change in adipocyte phenotype likely led to reduced release of adiponectin and enhanced release of IL-6^27^. Macrophage infiltration and crown-like structures were more abundant in VAT than SAT despite the fact that VAT cells were smaller than SAT cells. This finding extend to humans the observation – made in genetically obese mice^7^ – that the “critical death size” is lower in VAT adipocytes^9^, and that hypoxia induces adipose tissue remodeling^28^. Thirdly, VAT area and morphological alterations (CLS and inflammatory/necrotic changes) prevailed in T2D as compared to ND obese patients and correlated with beta-cell dysfunction. The latter result coupled with the excess fat infiltration of skeletal muscle indicates that the inherent beta-cell defect of T2D – as seen in lean T2D patients – is worsened by more severe VAT inflammation.

Of note is that cyst-like structures (in SAT from 2 T2D patients out of 26 examined) have not been previosuly described in fat tissues of lean or obese mice or in humans. Their architecture suggests that these structures may originate from extremely large, necrotic adipocytes^23^. It is noteworthy that the adipocytes in these two patients were particularly enlarged, exceeding the size of other obese patients by about 30%. In such cells, the very thin cytoplasm (thinnest cytoplasmic rims were about 1/10 of that of adipocytes of control patients) can rupture, thereby expelling lipid droplets into the interstitium. The resulting coalesced “oil” vacuole acts as a foreign body and is therefore surrounded by macrophages and giant multinucleated cells, in a manner similar to the mechanism hypothesized for CLS formation^8^ (Supplemental Figure S1). In line with this hypothesis, oil injections into the subcutaneus fat of mice give rise to similar structures (Cinti *et al*., personal observations).

Surgery provided a robust test for the cause-effect value of the histological/metabolic relationships. With major weight loss, all of the metabolic abnormalities improved despite the fact that in most patients BMI remained in the obese range. Thus, hyperinsulinemia and fasting insulin hypersecretion abated, dyslipidemia reversed, and the circulating concentrations of MCP-1 and IL-6 (but not TNFα) decreased. Insulin sensitivity of glucose metabolism and lipolysis improved in rough proportion to the amount of weight lost but remained subnormal in both ND and T2D subjects. In parallel with these metabolic changes, tissue histology was drastically altered^29, 30^. In subcutaneous fat, adipocyte size was reduced by ~50%, and CLS became rare. These results in postsurgery patients are consistent with recent data showing that adipose tissue morphology and insulin sensitivity in humans are closely linked^31–33^. Signs of acute intravascular inflammation almost disappeared together with a general restoration of adipocyte morphology. Certain stress-related changes persisted, however. Thus, accumulation of glycogen and collagen fibrils, dilated RER, crystals, alterations of basal membrane, and signs of degeneration in adipocytes, and sparse macrophages (mainly in T2D patients) were still present in SAT, possibly accounting for the persistence of circulating TNFα levels.

Collectively, the postsurgery results support the interpretation that obesity itself was a partially reversible cause of both the histopathology and the metabolic derangements of our patients. A circumstance takes exception to this conclusion, namely, that baseline beta-cell function was within the normal range in the ND subjects despite the fat and muscle alterations and, on the other hand, in T2D subjects beta-cell function was not fully restored after weight loss despite the almost recovery of SAT and muscle morphological alterations^29, 30^. The lack of VAT tissue after weight reduction in ours patients represents a limitation of our study as we cannot gauge the contribution of residual inflammatory/necrotic changes in VAT to beta-cell dysfunction. However, it is noteworthy that, in the baseline dataset VAT adipocyte area is reciprocally associated with beta-cell function (Table 3), and the association persists after adjusting for sex, SAT adipocyte area and intramyocellular fat (*p* = 0.002). On these grounds, it may be hypothesized that abnormalities in VAT may be more specifically linked with beta-cell dysfunction, possibly through residual inflammation as signaled by the unchanged levels of TNF-a and MCP-1 (Supplemental Table S1).

It is pertinent to recall that in non-diabetic patients omentectomy in patients undergoing bariatric surgery did not enhance the effect of bariatric surgery on insulin sensitivity but was associated with improved insulin secretion^34^. Therefore, the current results are compatible with the concept that diabetes is a condition of *intrinsic* beta-cell incompetence, which fat/muscle pathology may aggravate by propagation of inflammatory reactions^35–37^. The complex cross-talk between muscle^38^ and/or fat^39, 40^ and the islets of Langerhans is only beginning to be unraveled. The crosstalk between adipose tissue and the endocrine pancreas is also mediated by two circulating adipokines, namely adiponectin and adipsin. These factors, which are reduced in obesity models, may exert a direct action on beta-cell function and/or insulin sensitivity^41–43^. In line with this possibility, in our postsurgery patients the increased adiponectin levels may have contributed to the improvement of glucose metabolism.

The finding that neither body weight nor insulin sensitivity were restored to normal one year after surgery in either of our two study groups is the rule for severely obese subjects^14^; as a consequence, proof that the sequence starting with weight gain can be fully reversed is lacking.

In summary, in morbid obesity subcutaneous fat morphological alterations – adipocyte hypertrophy, degenerative changes and necrosis, macrophage infiltration and formation of crown-like/cyst-like structures – and muscle fat accumulation correlate with insulin resistance of glucose utilization and lipolysis and with raised plasma inflammatory biomarkers. Abnormalities of VAT, more than SAT, cellular changes and intramyocellular fat accumulation are related to impaired beta-cell function. Following surgery-induced weight loss, most of the subcutaneous fat and muscle changes resolve and metabolic functions improve. In the face of only subtle, residual pathological changes in adipose tissue, however, insulin sensitivity remains subnormal and, in T2D patients, beta-cell function is not fully restored, suggesting persistence of a degree of visceral fat abnormalities that deserves further study.

## Electronic supplementary material

Supplemental Table S1

Supplemental Figure S1
