# Publisher Correction: Muscle and adipose tissue morphology, insulin sensitivity and beta-cell function in diabetic and nondiabetic obese patients: effects of bariatric surgery

**DOI:** 10.1038/s41598-018-25221-1

**Published:** 2018-05-22

**Authors:** Stefania Camastra, Alessandra Vitali, Marco Anselmino, Amalia Gastaldelli, Rosario Bellini, Rossana Berta, Ilenia Severi, Simona Baldi, Brenno Astiarraga, Giorgio Barbatelli, Saverio Cinti, Ele Ferrannini

**Affiliations:** 1 Department of Clinical & Experimental Medicine, University of Pisa, Pisa, Italy; 2 Department of Experimental and Clinical Medicine-Center of Obesity, University of Ancona, Ancona, Italy; 3 Bariatric Surgery Unit, Santa Chiara Hospital, Pisa, Italy; 4 CNR Institute of Clinical Physiology, Pisa, Italy

Correction to: *Scientific Reports* 10.1038/s41598-017-08444-6, published online 21 August 2017

In this Article, the rows in Table 3 are misaligned. The correct Table 3 appears below.

**Table 3:** Correlation among clinical, histological, and metabolic parameters in the combined ND and T2D groups.

	BASELINE				BEFORE AND AFTER SURGERY											
	Adipocyte area VAT		CLS VAT		Adipocyte area SAT		CLS SAT		Perivascular muscle fat		Interfibrillar muscle fat		Intramyocellular fat		Adipocyte mitochondria size	
	**r**	***p***	**r**	***p***	**r**	***p***	**r**	***p***	**r**	***p***	**r**	***p***	**r**	***p***	**r**	***p***
Adipocyte area SAT	0.76	<0.0001		ns	—	—	0.60	<0.0001		ns	0.54	0.0001	0.57	0.0006	−0.36	0.01
CLS SAT	0.59	0.008	0.61	0.006	0.60	<0.0001	—	—		ns		ns	0.39	0.03		ns
Perivasc. muscle fat		ns	0.47	0.04		ns		ns	—	—	0.43	0.007		ns		ns
Interfibrill. muscle fat		ns		ns	0.54	0.001		ns	0.43	0.007	—	—	0.50	0.003		ns
Intramyocellular fat	0.60	0.005		ns	0.57	0.0006	0.39	0.03		ns	0.50	0.003	—	—		ns
BMI		ns		ns	0.89	<0.0001	0.50	0.001		ns	0.45	0.004	0.42	0.008	−0.61	<0.0001
Waist circumference	0.59	0.01	0.54	0.03	0.86	<0.0001	0.61	0.001	0.44	0.03	0.44	0.03	ns	ns	−0.41	0.004
Fasting glucose		ns	0.60	0.002	0.44	0.003	0.36	0.04	0.36	0.04		ns	0.35	0.04		ns
Fasting insulin		ns		ns	0.70	<0.0001	0.52	0.0008	0.46	0.004		ns	0.36	0.03	−0.20	0.049
HbA1c		ns	0.65	0.02	0.54	0.02	0.57	0.02		ns		ns		ns		ns
Mean Glucose	0.43	0.04	0.53	0.007	0.40	0.009	0.57	0.0003		ns	0.41	0.01		ns		ns
Adiponectin		ns		ns	−0.56	0.0002	−0.39	0.02		ns		ns		ns	0.54	0.0004
MCP1		ns		ns	0.35	0.03	0.37	0.03		ns		ns	0.40	0.02		ns
IL-6		ns		ns	0.50	0.001		ns	0.41	0.02		ns	0.40	0.02		ns
M		ns	−0.42	0.03	−0.62	<0.0001	−0.42	0.009	−0.35	0.03		ns		ns		ns
M/I		ns		ns	−0.72	<0.0001	−0.43	0.008	−0.35	0.03	−0.36	0.03		ns	0.38	0.004
AT-IR		ns		ns	0.58	<0.0001	0.35	0.04	0.51	0.001		ns		ns		ns
ß-GS	−0.58	0.004	−0.50	0.02		ns	−0.43	0.01		ns		ns	−0.48	0.004		ns
Fasting ISR		ns		ns	0.54	0.0002	0.45	0.006	0.41	0.002		ns		ns		ns

Additionally, this Article contains errors in the legend of Figure 4.

“In D and C electron microscopy of subcutaneous adipose tissue (SAT) of two obese patients with type 2 diabetes.”

should read:

“In D and E electron microscopy of subcutaneous adipose tissue (SAT) of two obese patients with type 2 diabetes.”

