# Supplementary material for: Muscle and adipose tissue morphology, insulin sensitivity and beta-cell function in diabetic and nondiabetic obese patients: effects of bariatric surgery
Source: Sci Rep. 2017 Aug 21;7:9007. doi: 10.1038/s41598-017-08444-6 (PMC5566429; doi:10.1038/s41598-017-08444-6)
Supplement: Supplementary file 1 — Supplemental Table S1 [file 41598_2017_8444_MOESM1_ESM.pdf]

Muscle and adipose tissue morphology, insulin sensitivity and beta-cell function in diabetic and nondiabetic obese patients: effects of bariatric surgery.

Camastra S*, Vitali A, Anselmino M, Gastaldelli A, Bellini R, Berta R, Severi I, Baldi S, Astiarraga B, Barbatelli G, Cinti S, Ferrannini E.

Supplemental Table S1 – Changes in anthropometric and metabolic parameters.

	ND			T2D		
	**Baseline**	***p*§**	**1 year**	**Baseline**	***p*§**	**1 year**
Number (F/M)	8 (7/1)		8 (7/1)	11 (7/4)		11 (7/4)
Body weight (kg)	140 ± 8	0.01	97 ± 6	138 ± 8	0.003	92 ± 5
BMI ($kg \cdot m^{-2}$)	50.4 ± 2.0	0.01	34.9 ± 1.4	50.5 ± 2.6	0.003	33.8 ± 1.6
FM (%)	50.4 ± 1.9	0.01	39.5 ± 2.1	46.2 ± 2.6	0.003	33.1 ± 2.8
FM (kg)	69.9 ± 5.2	0.01	38.2 ± 3.1	63.6 ± 5.6	0.003	31.0 ± 3.5
FFM (kg)	69.8 ± 4.7	0.01	58.5 ± 4.3	73.9 ± 5.9	0.008	61.1 ± 3.3
Fasting glucose (mmol/l)	5.7 ± 0.1	0.01	4.9 ± 0.1	9.1 ± 0.9	0.003	5.4 ± 0.2*
Fasting insulin (pmol/L)	130 [116]	0.01	45 [22]	137 [126]	0.003	49 [26]
Plasma NEFA (mmol/L)	0.61 [0.26]	ns	0.57 [0.28]	0.67 [0.11]	ns	0.68 [0.07]
Total cholesterol (mmol/L)	4.6 [1.2]	ns	4.1 [0.9]	4.3 [0.9]	ns	4.0 [0.9]
Triacylglycerols (mmol/L)	1.3 [1.1]	0.03	0.8 [0.5]	1.3 [0.6]	0.02	1.0 [0.1]
HDL-cholesterol (mmol/L)	1.3 [0.1]	ns	1.5 [0.1]	1.1 [0.1]	ns	1.1 [0.1]
Adiponectin (ng/ml)	6.5 ± 1.0	0.02	11.2 ± 1.8	6.1 ± 1.6	0.01	13.0 ± 3.2
MCP-1 (pg/ml)	250 [144]	0.05	161 [57]	271 [147]	ns	270 [129]
IL-6 (pg/ml)	3.49 [2.12]	0.05	1.68 [1.28]	6.09 [4.32]	0.01	2.21 [1.63]
TNFα (pg/ml)	1.10 [0.58]	ns	1.00 [0.58]	1.18 [0.50]	ns	1.09 [0.43]
M/I ($nmol \cdot min^{-1} \cdot kg_{FFM}^{-1} \cdot pM^{-1}$)	38.3 [24.7]	0.01	69.5 [22.9]	23.7 [26.7]	0.003	87.6 [41.3]
RaGly ($\mu mol \cdot min^{-1}$)	353 [206]	ns	227 [142]	349 [54]	0.03	275 [113]
AT-IR ($mmol \cdot min^{-1} \cdot kg_{FM}^{-1} \cdot pM$)	0.53 [1.17]	0.05	0.26 [0.25]	0.86 [0.93]	0.03	0.44 [0.26]
Mean Glucose (mmol/L)	6.0 ± 0.2	ns	5.9 ± 0.1	9.3 ± 0.9	0.003	6.4 ± 1.0*
ß-GS ($pmol \cdot min^{-1} \cdot m^{-2} \cdot mM^{-1}$)	138 [50]	0.02	92 [18]	27 [24]	0.007	64 [38]*
Total insulin output ($nmol \cdot m^{-2}$)	86 [32]	ns	68 [29]	57 [43]	ns	59 [30]
Fasting ISR ($pmol \cdot min^{-1} \cdot m^{-2}$)	140 [80]	0.02	63 [21]	100 [80]	0.05	70 [29]

§ *p* for the changes at 1 year *vs* baseline by Wilcoxon sign rank test; * $p \leq 0.05$ for the between-group difference at 1 year.
