# Supplementary material for: Muscle and adipose tissue morphology, insulin sensitivity and beta-cell function in diabetic and nondiabetic obese patients: effects of bariatric surgery
Source: Sci Rep. 2017 Aug 21;7:9007. doi: 10.1038/s41598-017-08444-6 (PMC5566429; doi:10.1038/s41598-017-08444-6)
Supplement: Supplementary file 2 — Supplemental Figure S1 [file 41598_2017_8444_MOESM2_ESM.pdf]

Muscle and adipose tissue morphology, insulin sensitivity and beta-cell function in diabetic and nondiabetic obese patients: effects of bariatric surgery.

Camastra S*, Vitali A, Anselmino M, Gastaldelli A, Bellini R, Berta R, Severi I, Baldi S, Astiarraga B, Barbatelli G, Cinti S, Ferrannini E.

Supplemental Figure S1 – Hypothesis for cyst-like structure formation.

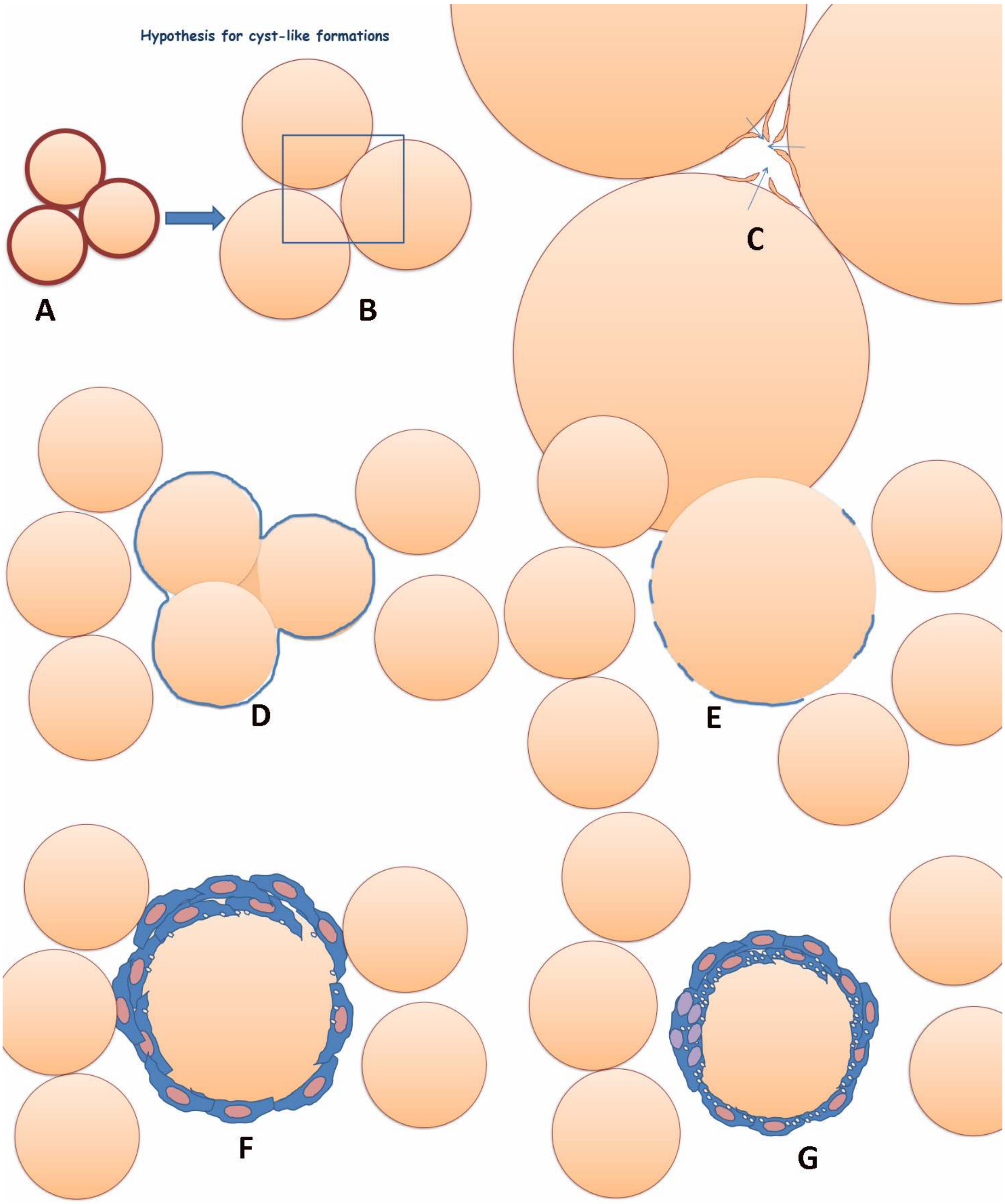

Supplemental Figure S1 – Hypothesis for cyst-like structure formation. **A**. Normal adipocytes with normal cytoplasmic rim (red line), **B**. In obese adipose tissue, hypertrophic adipocytes are in close association and show a very thin cytoplasmic rim (thin red line), **C**. Some of the largest hypertrophic adipocytes present a convergent rupture of the thin cytoplasmic rim (arrows), **D**. The lipid droplets of damaged adipocytes coalesce to form a unique large, oil-like droplet surrounded by remnants of cytoplasmic rim (dotted blu line), **E**. The oil-like droplet is surrounded by macrophages which start the process of reabsorption (**F**), with formation of multinuclear giant cells (**G**).
